# Pre-hypertension: another ‘pseudodisease’?

**DOI:** 10.1186/1741-7015-11-211

**Published:** 2013-09-25

**Authors:** Pascal Meier, Franz H Messerli, Andreas Baumbach, Alexandra J Lansky

**Affiliations:** 1 The Heart Hospital, University College London Hospitals UCLH, 16-18 Westmoreland Street, London W1T 7HA, UK; 2 Division of Cardiology, Yale Medical School, New Haven, CT, USA; 3 St Luke’s Roosevelt Hospital, Columbia University College of Physicians and Surgeons, New York, NY 10019, USA; 4 Bristol Heart Institute, Bristol, UK

**Keywords:** Hypertension, Pre-hypertension, Prevention, Cardiovascular

## Abstract

Hypertension is one of the most important and common cardiovascular risk factors. Defining the level at which blood pressure starts causing end-organ damage is challenging, and is not easily answered. The threshold of blood pressure defining hypertension has progressively been reduced over time, from systolic >160 mmHg to >150 mmHg, then to >140 mmHg; and now even blood pressures above 130 to 120 mmHg are labeled as ‘pre-hypertension’ by some expert committees. Are interest groups creating another ‘pseudodisease’ or is this trend scientifically justified? A recent meta-analysis published in *BMC Medicine* by Huang *et al*. clearly indicates that pre-hypertension (120 to 140/80 to 90 mmHg) is a significant marker of increased cardiovascular risk. This raises the question as to whether we now need to lower the threshold of ‘hypertension’ (as opposed to ‘pre-hypertension’) to >120/80 mmHg, redefining a significant proportion of currently healthy people as ‘patients’ with an established disease. These data need to be interpreted with some caution. It is controversial whether pre-hypertension is an independent risk factor or just a risk marker and even more controversial whether treatment of pre-hypertension will lower cardiovascular risk.

Please see related research: http://www.biomedcentral.com/1741-7015/11/177.

## Introduction

The systematic review by Huang *et al*. addresses a very important public health topic [1]. What exactly is hypertension? The English clergyman Stephen Hales was the first to describe and define ‘blood pressure’ circa 1730 when he measured the rise in a column of blood in a glass tube bound into an artery. Measuring blood pressure non-invasively was first performed in 1854 by the German physician Karl Vierordt, and later in a simplified manner by the Italian physician Scipione Riva-Rocci in 1896. Measuring blood pressure is one of the oldest and remains the most common medical examinations. Nevertheless, the absolute blood pressure threshold to define hypertension remains controversial, as we still do not fully understand at what level high blood pressure becomes a ‘disease’ and cardiovascular risk factor (Table 1).

**Table 1:** Definitions and thresholds from various professional bodies

**Professional body**	**Classifications**	**Systolic values (mmHg)**	**Diastolic values (mmHg)**
European Society of Cardiology (ESC), 2013	Optimal	<120	<80
	Normal	120 to 129	80 to 84
	High normal	130 to 139	85 to 89
	Grade I hypertension	140 to 159	90 to 99
	Grade II	160 to 179	100 to 109
	Grade III	>179	>109
	Isolated systolic hypertension	>139	<90
Joint National Committee (JNC 7), 2003	Normal	<120	<80
	Pre-hypertension	120 to 139	80 to 89
	Hypertension	≥140	≥90
National Institute for Health and Care Excellence (NICE), 2011	Normal	<140	<90
	Stage I hypertension	≥140	≥90
	Stage II hypertension	≥160	≥100
	Severe hypertension	≥180	≥110

As illustrated by these variable definitions from different committees, it is difficult to come up with a clear cut-off value to define ‘good’ and ‘bad’. While clinicians understandably prefer clear binary disease definitions, there is a continuous association between blood pressure and cardiovascular risk [2].

In a recent study published in *BMC Medicine*, Huang *et al*. challenge current guidelines which label blood pressures >140/>90 mmHg as being ‘normal’ by concluding that pre-hypertension (even at lower levels of 120 to 129/80 to 84 mmHg) is a significant cardiovascular risk factor.

### New evidence

Huang *et al*. have performed a systematic review on the clinical relevance of pre-hypertension [1]. They performed a pooled analysis of 18 prospective cohort studies including a total of 468,561 patients. Patients with pre-hypertension had a 55% increased risk for cardiovascular disease, a 50% increased risk for coronary artery disease, and a 71% increased stroke risk (RR 1.71 (95% confidence interval 1.55 to 1.89)). The authors went a step further and subdivided the pre-hypertension group into ‘low-range pre-hypertension’ and ‘higher-range pre-hypertension’. Even in the low-range pre-hypertension cohort, defined by blood pressures of 120 to 129/80 to 84 mmHg, the risk of cardiovascular disease was 46% higher than for individuals with normal blood pressures (RR 1.46 (1.32 to 1.62)). Therefore, according to this very large analysis, even lower-range pre-hypertension has a significant impact on morbidity and mortality.

Let us now have a closer look at some individual studies included in this meta-analysis. The Framingham Heart Study (6,859 participants who were initially free of hypertension and cardiovascular disease) showed a 2.5-fold increased risk for cardiovascular events at 10 years for women and a 1.6-fold increase for men with blood pressures between 130 to 139/85 to 89 mmHg [3]. In line with the meta-analysis of Huang *et al*., the Framingham study further showed an increased risk even for those with blood pressure values of 120 to 129/80 to 84 mmHg compared with those with ‘optimal’ blood pressure (<120/<80 mmHg). Another very important study in this context is the Women's Health Initiative, involving over 60,785 women in their postmenopausal phase. Pre-hypertensive women showed a 76% increased risk for cardiovascular death, a 93% increased risk for myocardial infarction and 36% increased risk for stroke [4]. One endpoint that was not assessed in these studies was the impact of pre-hypertension on one important end organ: the kidneys. One study that did assess pre-hypertension and microalbuminuria as an early sign of kidney dysfunction showed an increased incidence compared to patients with optimal blood pressure [5]. This finding further supports the findings of Huang *et al*.

The question arises based on these data, whether we should now reclassify ‘pre-hypertension’ as ‘hypertension’, even at the low blood pressure (BP) range, which would apply to a significant proportion of the population as newly labeled hypertensive patients? It is estimated that about 37% of people in the US, that is, more than 100 million fall within this pre-hypertension category. Whether these patients would benefit from pharmacologic therapy and if so, what the exact cost/benefit relation would be remains uncertain. Pre-hypertension is clearly associated with cardiovascular risk, but it is questionable whether a blood pressure level in this pre-hypertension range is a causal risk factor for cardiovascular events *per se*. We know that pre-hypertensive individuals are much more likely to progress to hypertension. In the Framingham cohort, patients with ‘higher range pre-hypertension’ (130 to 139/85 to 89 mmHg) developed hypertension in 37% of cases, those with ‘lower range pre-hypertension’ developed hypertension in 18% of cases and those with ‘optimal blood pressure’ only in 5% of cases, over a 4-year period in patients >65 years [3]. Of course, the long-term cardiovascular event rate is higher in those people, and this may not be related to their history of pre-hypertension. Beyond that, there are probably other ‘confounding factors’. Even though most studies used multivariate adjustments to reduce confounding bias, such adjustments can only be made for available measured variables. It is likely that there are other factors which were not measured and which independently influence blood pressure and cardiovascular outcomes, such as environmental and genetic factors.

### Therapeutic implications

The question still remains whether active treatment of pre-hypertension will prevent progression to more advanced hypertension and future cardiovascular events. Experimental data indicate that it may actually be possible to change the natural history of pre-hypertension. In a hypertensive rat model, if antihypertensive therapy is given within the first 2 to 6 weeks of life it can prevent the development of hypertension [6].

In the clinical setting, these findings could not be fully confirmed. The ‘TRial of Preventing Hypertension’ (TROPHY) study was a 4-year trial of 806 patients with pre-hypertension who were randomly assigned to 2 years of therapy with either candesartan or placebo (16 mg/day) [7]. After 2 years, all patients continued therapy with placebo for another 2 years. At 2 years, the systolic and diastolic pressures were significantly lower with candesartan therapy compared to placebo. However, within 9 months of cessation of candesartan therapy, the pressures rose to values similar to those in the placebo group. Similarly, in the Medical Research Council trial in nearly 3,000 participants using thiazide diuretic or beta blockers versus placebo, the blood pressures dropped during therapy, but within 6 months after cessation, reached the levels of the placebo group [8].

While temporary treatment of pre-hypertension does not seem to prevent progression to hypertension, the question remains as to whether it reduces CV risk? Data on this question are conflicting. A recent meta-analysis pooling the results of trials of antihypertensive treatment in patients with cardiovascular disease but without hypertension showed a benefit. It was based on 25 randomized trials and 64,162 participants and found a 23% reduction in the risk of stroke, a 20% reduced risk of myocardial infarction, a 15% reduced risk of heart failure and a 13% reduced mortality risk [9]. Whether this also applies to individuals without manifest cardiovascular disease is unclear.

### Ambulatory blood pressure measurement (ABPM)

ABPM has become a useful tool to evaluate the true blood pressure burden and it has been shown to be a better predictor of cardiovascular events than are office blood pressure measurements. In a recent study of almost 5,000 patients from the Spanish ABPM Registry 60% of patients with office pressure of 130 to 139/85 to 89 mmHg, 42% with office pressure of 140 to 159/90 to 99 mmHg and 53% with office blood pressures of above 160/100 mmHg were actually normotensive according to the 24-h ABPM measuring criteria, that is, their mean ABPM was below 130/80 mmHg) [10]. Clearly therefore office blood pressure measurements can be deceptive in identifying the specific stage of hypertensive cardiovascular disease. This problem was also encountered in a very recent analysis of Mahfoud *et al*., in patients who had renal denervation on ABPM. There was a significant reduction of 8 to 10 mm Hg systolic and 4 to 7 mm Hg diastolic, but clearly less impressive that the reduction of the office blood pressure in the same population (21 to 27 mm Hg (systolic)) and (9 to 12 mm (diastolic)) [11].

## Conclusions

The data of Huang *et al*. clearly indicate that a blood pressure level currently defined as pre-hypertension is a significant marker of increased cardiovascular risk. However, we lack evidence that pre-hypertensive blood pressure itself is harmful and that an earlier intervention to reduce the blood pressure is beneficial in the absence of cardiovascular disease. Since pre-hypertensive individuals are at a high risk to progress to sustained hypertension, we advise periodic screening.

## Competing interests

FHM: *ad hoc* consultant for the following organizations: Novartis, Daiichi Sankyo, Pfizer, Takeda, Abbott, Servier, Medtronic, Ipca Laboratories Ltd.

## Authors’ contributions

PM drafted the first version, FHM, AB and AJL revised it critically for its intellectual content. All authors contributed substantially to this editorial and approved the final manuscript.

## Authors’ information

Pascal Meier is a cardiologist at University College London, UK, and Yale Medical School, CT, USA (http://www.drpascalmeier.com). He is editor-in-chief of the journal Open Heart and associate editor of the Cochrane Heart group (http://heart.cochrane.org/). Franz H Messerli is an expert in hypertension. He is Professor of Clinical Medicine at Columbia University College of Physicians and Surgeons, New York. Andreas Baumbach is a cardiologist at the Bristol Heart Institute. He has an interest in novel treatment options for hypertension, such as renal artery denervation. He is a co-director of the EuroPCR course, one of the major international cardiology conferences. Alexandra Lansky is a cardiologist and associate professor at Yale University School of Medicine and a renowned expert in clinical cardiovascular research. She is heading the Yale-UCL Cardiovascular Research collaborative (http://www.yale-ucl.org/index.aspx).

## Pre-publication history

The pre-publication history for this paper can be accessed here:

http://www.biomedcentral.com/1741-7015/11/211/prepub
